# TRIC-A Loss Sensitizes the Heart to β-Adrenergic Stress and Drives Cardiomyocyte Death and Fibrosis

**DOI:** 10.3390/biom16020181

**Published:** 2026-01-23

**Authors:** Ki Ho Park, Daiju Yamazaki, Xinyu Zhou, Shinji Komazaki, Chengzhu Zhao, Miyuki Nishi, Jingsong Zhou, Hiroshi Takeshima, Jianjie Ma

**Affiliations:** 1 Department of Surgery, Division of Surgical Sciences, University of Virginia, Charlottesville, VA 22903, USA; kiho.park@virginia.edu (K.H.P.); ywx5wp@virginia.edu (X.Z.); 2 Research Unit for Physiological Chemistry, the Center for the Promotion of Interdisciplinary Education and Research, Kyoto University, Kyoto 606-8502, Japan; daiju-y@nihs.go.jp (D.Y.);; 3 Department of Biological Chemistry, Graduate School of Pharmaceutical Sciences, Kyoto University, Kyoto 606-8501, Japannishi.miyuki.3a@kyoto-u.ac.jp (M.N.); 4 Division of Pharmacology, National Institute of Health Sciences, Kawasaki 210-9501, Japan; 5 Department of Anatomy, Saitama Medical University, Saitama 350-0495, Japan; 6 Department of Kinesiology, University of Texas at Arlington, Arlington, TX 76010, USA

**Keywords:** TRIC-A, sarcoplasmic reticulum, calcium handling, β-adrenergic stimulation, ryanodine receptor 2, mitochondrial Ca^2+^ overload, cardiomyocyte necrosis, cardiac fibrosis

## Abstract

Trimeric intracellular cation channel A (TRIC-A) provides counter-ion support for sarcoplasmic reticulum (SR) Ca^2+^ release, yet its physiological role in the intact heart under stress remains poorly defined. Here, we demonstrate that TRIC-A is essential for maintaining balanced SR Ca^2+^ release, mitochondrial integrity, and cardiac resilience during β-adrenergic stimulation. *Tric-a^−/−^* cardiomyocytes exhibited Ca^2+^ transients evoked by electrical stimuli and exaggerated isoproterenol (ISO)-evoked Ca^2+^ release, consistent with SR Ca^2+^ overload. These defects were accompanied by selective upregulation of protein kinase A (PKA)-dependent phosphorylation of ryanodine receptor 2 (RyR2) (S2808) and phospholamban (PLB) (S16). Acute ISO challenge induced mitochondrial swelling, cristae disruption, and Evans Blue Dye uptake, and elevated circulating troponin T in *Tric-a^−/−^* hearts, hallmarks of necrosis-like cell death. Mitochondrial Ca^2+^ uptake inhibition with Ru360 markedly reduced membrane injury, establishing mitochondrial Ca^2+^ overload as the proximal trigger of cardiac cell death. With sustained β-adrenergic stimulation by ISO, *Tric-a^−/−^* hearts developed extensive interstitial and perivascular fibrosis without exaggerated hypertrophy. Cardiac fibroblasts lacked TRIC-A expression and displayed normal Ca^2+^ signaling and activation, indicating that fibrosis arises secondarily from cardiomyocyte injury rather than fibroblast-intrinsic abnormalities. These findings identify TRIC-A as a critical regulator of SR-mitochondrial Ca^2+^ coupling and a key molecular safeguard that protects the heart from catecholamine-induced injury and maladaptive remodeling.

## 1. Introduction

Cellular Ca^2+^ handling is fundamental to cardiac excitation–contraction coupling [1,2,3,4,5,6]. During each heartbeat, tightly coordinated Ca^2+^ release from the sarcoplasmic reticulum (SR) through ryanodine receptor 2 (RyR2) activates the contractile machinery, while subsequent reuptake restores SR Ca^2+^ load for the next cycle [7,8,9,10]. Because Ca^2+^ efflux from the SR is electrogenic, a complementary counter-ion movement is required to stabilize SR membrane potential and preserve efficient release [11,12]. Trimeric intracellular cation (TRIC) channels, K^+^-permeable pores embedded in the SR/ER membrane, have emerged as leading candidates for providing this counter-current [13,14,15,16].

Mammals possess two TRIC isoforms: TRIC-B, which is broadly distributed among cell types, and TRIC-A, which is enriched in excitable cells including skeletal muscle, smooth muscle, and the heart [13,17]. Genetic ablation studies have revealed distinct physiological roles for these isoforms [13,18,19]. TRIC-B deletion impairs IP_3_ receptor-dependent Ca^2+^ signaling and causes perinatal respiratory failure due to dysfunctional alveolar epithelial cells, underscoring its essential role in ER Ca^2+^ homeostasis [18]. In contrast, loss of TRIC-A yields tissue-specific defects linked to RyR-dependent Ca^2+^ release [15,19,20]. *Tric-a*-null skeletal muscle exhibits reduced Ca^2+^ spark frequency, SR Ca^2+^ overload, and stress-induced contractile alternans [21], whereas in vascular smooth muscle, TRIC-A deficiency impairs Ca^2+^ spark generation and amplifies IP_3_ receptor-mediated Ca^2+^ transients, leading to hypertension [15]. In the developing heart, combined deletion of *Tric-a* and *Tric-b* results in embryonic lethality, highlighting the non-redundant importance of TRIC channels for Ca^2+^ handling in immature cardiomyocytes [13]. These isoform-specific roles are further reflected in human disease; loss of TRIC-B, either through *TRIC-B* mutations in humans or *Tric-b* knockout in mice, causes bone defects resembling osteogenesis imperfecta [22,23,24,25,26]. In parallel, variants in *TMEM38A* (e.g., *TRIC-A*) have been reported in patients with muscle disorders, including Emery–Dreifuss muscular dystrophy [27,28,29].

In adult cardiomyocytes, TRIC-A loss suppresses spontaneous Ca^2+^ sparks but exaggerates global caffeine-evoked release, suggesting an imbalance between local signaling events and overall SR discharge [19,20]. Mechanistically, TRIC-A has been shown to physically associate with RyR2 via its C-terminal domain, increasing RyR2 open probability and fine-tuning local Ca^2+^ release dynamics [19]. These observations support a model in which TRIC-A serves dual functions: (a) providing counter-ion flux to support robust RyR2-mediated Ca^2+^ release, and (b) directly modulating RyR2 gating to prevent store-overload-induced Ca^2+^ release (SOICR) [19,20]. In the absence of this regulatory influence, cardiomyocytes become prone to spontaneous Ca^2+^ waves, arrhythmogenic signaling, and subsequent cellular stress [19,30].

Despite its recognized importance in SR Ca^2+^ regulation, the role of TRIC-A in maintaining cardiac resilience under neurohumoral stress remains poorly defined. β-adrenergic stimulation, a central regulator of the physiological “fight-or-flight” response, enhances RyR2 activity and increases SR Ca^2+^ turnover to boost cardiac output [31]. However, chronic or excessive β-adrenergic drive destabilizes SR Ca^2+^ homeostasis, promotes mitochondrial dysfunction, and contributes to cardiomyocyte death, fibrosis, and heart failure [32,33,34]. Whether TRIC-A acts as a molecular safeguard that stabilizes SR Ca^2+^ release during β-adrenergic stress has not been addressed.

Since the discovery of TRIC channels in 2007 [13], advances in structural biology and molecular modeling have deepened understanding of their functional architecture [35,36,37,38]. Crystal structures published in 2016 confirmed the trimeric organization of the channel and provided evidence of potential interaction interfaces with RyR complexes [14,19,37]. These structural insights, combined with emerging data on SR/ER-mitochondrial Ca^2+^ crosstalk, underscore how TRIC channel dysfunction may influence metabolic stress responses and contribute to cardiac pathophysiology [39]. Here, we investigate the role of TRIC-A in maintaining cardiomyocyte stability under β-adrenergic stimulation using complementary in vitro and in vivo models of TRIC-A deficiency.

## 2. Materials and Methods

### 2.1. Animals

*Tric-a^−/−^* mice were generated as previously described and maintained on a C57BL/6J background with wild-type littermates as controls [13,19]. Male mice aged 8–12 weeks were used. Animals were housed under controlled temperature and light–dark cycles with ad libitum access to food and water. Protocols were approved by the Institutional Animal Care and Use Committee (IACUC) of the University of Virginia (Protocol #4410 approved on 28 October 2022). All experimental procedures were approved by the Animal Research Committee of Kyoto University ((Protocol #2012-10 approved on 28 February 2014)) according to the regulations on the animal experimentation of Kyoto University and the Institutional Animal Care and Use Committee and conformed to NIH guidelines.

### 2.2. In Vivo β-Adrenergic Stimulation

β-adrenergic stimulation was achieved by continuous administration of isoproterenol using subcutaneous osmotic minipumps (ALZET Osmotic Pump Model No. 1003D for acute stimulation or No. 2002 for chronic stimulation, ALZET LLC, Campbell, CA, USA). For chronic stimulation, isoproterenol hydrochloride (Sigma-Aldrich, St. Louis, MO, USA, Cat. No. 1351005) was dissolved in sterile saline and delivered via an osmotic minipump at a dose of 60 mg/kg/day for 14 days. Control mice received saline-filled minipumps. Minipumps were implanted under isoflurane anesthesia, and animals were monitored daily. For acute stimulation, isoproterenol (60 mg/kg/day) or isoproterenol plus Ru360 (50 nmol/kg/day, Merck, Burlington, MA, USA, Cat. No. 557440) were dissolved in sterile saline and delivered via an osmotic minipump for 1–3 days.

### 2.3. In Vivo Angiotensin II and Phenylephrine Treatment

To induce hypertrophic remodeling, angiotensin II (AngII) or phenylephrine (PE) was administered continuously using subcutaneous osmotic minipumps (ALZET, Model No. 2002) implanted in the dorsal region under isoflurane anesthesia. AngII was delivered at 2 mg/kg/day for 14 days, and PE was delivered at 75 mg/kg/day for 14 days. Control mice received saline-filled pumps. Animals were monitored daily throughout the infusion period.

### 2.4. Isolation of Ventricular Cardiomyocytes and Cardiac Fibroblasts

Ventricular cardiomyocytes and fibroblasts were isolated using a standard Langendorff perfusion protocol as previously described [19,40,41]. Isolated hearts from adult *Tric-a^−/−^* and WT littermate mice were perfused with a Langendorff apparatus at 37 °C. The enzyme digestion step consisted of perfusing Tyrode’s solution containing 0.4 mg/mL collagenase (Type II, ~250 U/mg; Worthington, Lakewood, NJ, USA, Cat. No. LS004176) and 0.1 mg/mL protease (Type XIV, Sigma-Aldrich, Cat. No. P5147) for 18 min. The Tyrode’s solution contained (in mM) 120 NaCl, 5.4 KCl, 1.2 NaH_2_PO_4_, 1.2 MgCl_2_, 5.6 glucose, 10 BDM and 1 taurine (pH 7.4). Cardiomyocytes were dissociated from digested ventricles by gentle mechanical dissociation and used within 3 h. Cardiac fibroblasts were also dissociated from ventricles by gentle mechanical dissociation and used within 5 passages. For cardiac fibroblast proliferation assay, cardiac fibroblasts (passages ≤ 5) were plated at an equal density in culture dishes and allowed to attach overnight. Cells were then treated with vehicle, isoproterenol (10 µM), or TGF-β (10 ng/mL). Proliferation was quantified by MTT assay (Dojindo, Kumamoto, Japan, Cat. No. CK04-01).

### 2.5. Ca^2+^ Imaging in Cardiomyocytes and Cardiac Fibroblasts

For intracellular Ca^2+^ cycling measurements, cardiomyocytes were loaded with 10 μM indo-1 (Thermo Scientific, Waltham, MA, USA, Cat. No. I1203) for 10 min, followed by 10 min de-esterification. Indo-1 was excited at 340 nm, and emission was collected at 405 nm and 485 nm. Cells were studied in a solution containing (in mM), 140 NaCl, 5.4 KCl, 1.8 CaCl_2_, 0.5 MgCl_2_, 10 HEPES, and 5.6 glucose (pH 7.4), under basal conditions and during stimulation with 100 nM isoproterenol. Cardiomyocytes were paced at 1 Hz using extracellular platinum electrodes. Fluorescence signals were expressed as F405/F485 and analyzed using MetaFlour ver7.7r3 (Molecular Devices, San Jose, CA, USA).

For monitoring intracellular Ca^2+^ concentration ([Ca^2+^]*_i_*) in cardiac fibroblasts (CFs), the isolated and passaged CFs were incubated with 5 μM Fura-2 AM (Dojindo, F015-10). Cells were alternately excited at 340 and 380 nm. A CCD camera (ImagEM, Hamamatsu Photonics, Hamamatsu, Japan) mounted on the microscope (DMI 4000B, Leica) equipped with a polychromatic illumination system (MetaFluor ver7.7r3) was used to capture fluorescence emission at >510 nm at room temperature. SR store content was assessed by ionomycin and thapsigargin-induced release, and SOCE by re-addition of extracellular Ca^2+^ after depletion.

### 2.6. Transmission Electron Microscopy

Transmission electron microscopy was performed as previously described. Left ventricular tissue was fixed in 2.5% glutaraldehyde in 0.1 M phosphate buffer, post-fixed in 1% osmium tetroxide, dehydrated through graded ethanol, and embedded in epoxy resin. Ultrathin sections (~80 nm) were stained with uranyl acetate and lead citrate and examined by transmission electron microscopy (JEM-200X, JEOL, Tokyo, Japan).

Mitochondrial morphology was analyzed from multiple randomly selected fields per heart by investigators blinded to genotype and treatment.

### 2.7. Serum Troponin T Assay

Blood was collected by cardiac puncture, centrifuged, and serum stored at −80 °C. Troponin T concentrations were measured by ECLIA (electrochemiluminescence immunoassay) at SRL, Inc. (Tokyo, Japan) using their routine clinical assay (test directory information provided by SRL).

### 2.8. Evans Blue Dye Uptake

Cardiomyocyte membrane integrity was assessed using Evans blue dye (EBD) uptake. Mice received an intraperitoneal injection of Evans blue dye (1% in phosphate-buffered saline, 10 μL/g body weight). Hearts were harvested 24 h after dye injection, rinsed in phosphate-buffered saline, embedded in optimal cutting temperature compound, and frozen. Serial 5–10 μm cryosections from the mid-ventricular region were examined by fluorescence microscopy using identical acquisition settings. EBD-positive cardiomyocytes were identified by intracellular red fluorescence and quantified from multiple non-overlapping fields. All analyses were performed in a blinded manner. For time-course experiments, Evans blue dye uptake was assessed after 1, 3, 7 and 14 days of isoproterenol treatment, as indicated.

### 2.9. Histology and Fibrosis Quantification

Hematoxylin and eosin (HE) staining and Masson trichrome (MT) staining were conducted by KAC (KAC Co., Ltd., Kyoto, Japan) using their standard protocols. Hearts were excised, rinsed briefly in ice-cold phosphate-buffered saline (PBS), and fixed in 10% neutral-buffered formalin at room temperature for 24 h. Fixed tissues were dehydrated through a graded ethanol series, cleared in xylene, and embedded in paraffin according to standard histological procedures. Transverse sections were cut at 5 μm thickness from the mid-ventricular level using a rotary microtome and mounted on glass slides. For assessment of myocardial fibrosis, sections were stained with Masson’s trichrome following the manufacturer’s instructions (Sigma-Aldrich, Cat. No. HT15). Collagen deposition was visualized as blue staining, whereas myocardium appeared red. Whole-section images were acquired using a bright-field microscope (BX53, EVIDENT, Nagano, Japan) equipped with a digital camera under identical acquisition settings for all samples. Fibrotic area was quantified using Fiji (ImageJ, NIH; version 1.54p) software. Collagen-positive (blue) regions were segmented using RGB color thresholding, using a fixed threshold across all images within an experiment.

### 2.10. Quantitative RT-PCR

For quantitative PCR analysis, total RNA extracted with Isogen (Nippon Gene, Tokyo, Japan) from CFs were used as templates for cDNA synthesis (ReverTra ACE qPCR-RT kit, Toyobo, Osaka, Japan) and analyzed using a real-time PCR system (Thermal Cycler Dice TP800, Ver. 3.01, Takara, Shiga, Japan) according to the manufacturer’s instructions. The cycle threshold (Ct) was determined from the cDNA amplification curve as an index for relative mRNA content in each reaction. PCR specificity was confirmed by agarose gel electrophoresis. The primer sequences are listed in Supplementary Table S1.

### 2.11. Western Blotting and Immunostaining

For Western blot, protein lysates were separated by SDS-PAGE, transferred to PVDF (Polyvinylidene fluoride) membranes, and probed with antibodies against Cav1.2 (Alomone Labs, Jerusalem, Israel, Cat. No. ACC-003), pRyR2 Ser2808 (Thermo Scientific, Cat. No. PA5-36758), pRyR2 Ser2814 (Thermo Scientific, Cat. No. PA5-104558), RyR2 (Thermo Scientific, Cat. No. MA3-925), IP3R2 (Santa Cruz, Dallas, TX, USA, Cat. No. sc-7278), NCX1 (homemade), pPLB Ser16 (Badrilla, Leeds, UK, Cat. No. A010-12AP), pPLB Thr17 (Badrilla, Cat. No. A010-13), PLB (Thermo Scientific, Cat. No. MA3-922), SERCA2 (Abcam, Cambridge, UK, Cat. No. ab2861), CSQs (Santa Cruz, Cat. No. sc-390999), JP2 (homemade), TRIC-A (homemade), TRIC-B (homemade), and Actin (Abcam, Cat. No. ab179467). Blots were visualized by chemiluminescence. Abbreviations are defined in the Abbreviations section below.

### 2.12. Statistical Analysis

The sample size (n) and the statistical tests used for each dataset are reported in the figure legends. Unless otherwise stated, n represents independent biological replicates (individual mice for in vivo experiments; independent cell preparations or isolations for primary cell studies). Data are presented as group means with error bars representing SEM, as indicated in the figure legends.

Normality was assessed using the Shapiro–Wilk test. For comparisons between two groups, a two-tailed unpaired Student’s *t* test was used for normally distributed data; otherwise, the Mann–Whitney U test was applied. For experiments involving two independent factors (e.g., genotype × treatment), two-way ANOVA with Tukey’s post hoc multiple-comparisons test was performed. For experiments involving three independent factors (e.g., genotype × treatment × time), three-way ANOVA was used, followed by Tukey’s multiple-comparisons test where appropriate, as indicated in the figure legends.

Homogeneity of variance was evaluated by visual inspection of residuals and group dispersion, and no gross variance inequality was observed. All statistical tests were two-tailed, and *p* < 0.05 was considered statistically significant. Statistical analyses were performed using GraphPad Prism (version 10.5.0).

No data points were excluded unless predefined technical failure criteria were met (e.g., unsuccessful isolation or inadequate signal quality precluding quantification). Any exclusions and final sample sizes are reported in the corresponding figure legends.

## 3. Results

### 3.1. TRIC-A Deficiency Leads to SR Ca^2+^ Overload and Baseline Hyperphosphorylation of RyR2 and Phospholamban

To define the role of TRIC-A in excitation–contraction coupling, we first assessed Ca^2+^ handling in ventricular cardiomyocytes under baseline and β-adrenergic stimulation. Cardiomyocytes were isolated from four mouse groups: WT, *Tric-a^−/−^*, and mice treated with or without isoproterenol (ISO).

**Figure 1:**
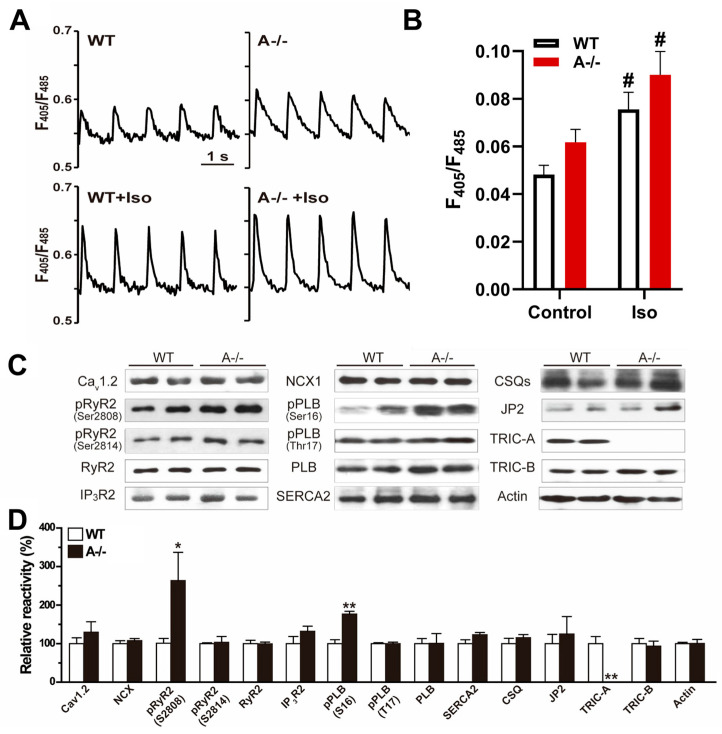
**TRIC-A stabilizes Ca^2+^ release and limits baseline phosphorylation of RyR2 and phospholamban.** (**A**) Representative Ca^2+^ transient traces in WT and *Tric-a^−/−^* cardiomyocytes during electrical stimulation with or without ISO. (**B**) Quantification of Ca^2+^ transient amplitude (*n* = 21). Two-way ANOVA revealed significant main effects of genotype (*p* < 0.05) and treatment (*p* < 0.001), with no significant interaction. Tukey’s multiple comparisons are indicated (# *p* < 0.05 Vs. Saline). (**C**) Immunoblots of Ca^2+^-handling proteins with actin control. (**D**) Hyperphosphorylation of RyR2 (S2808) and PLB (S16) in *Tric-a^−/−^* hearts (*n* = 4–8). Student *t*-test *, ** *p* < 0.05, 0.01. Original Western Blot images can be found in the Supplementary Materials.

When whole-cell Ca^2+^ transients were recorded during field stimulation, *Tric-a^−/−^* myocytes exhibited significantly larger peak amplitudes compared with WT cells (Figure 1A). ISO enhanced Ca^2+^ transient amplitude in both genotypes (Figure 1A,B). These findings are consistent with our previous observations and support a model in which loss of TRIC-A diminishes the efficiency of local RyR2-mediated Ca^2+^ release, causing progressive SR Ca^2+^ overload. Prior work by Zhou et al. demonstrated that *Tric-a^−/−^* cardiomyocytes generate fewer spontaneous Ca^2+^ sparks yet display higher spark amplitude, indicating that SR overload persists despite reduced local release frequency [19]. More recently, we confirmed that while spontaneous sparks are compromised, global caffeine-evoked Ca^2+^ release is markedly exaggerated in *Tric-a^−/−^* cardiomyocytes, further indicating excessive SR Ca^2+^ storage [20,39].

Despite substantial differences in Ca^2+^ handling at the functional level, the expression of major Ca^2+^-handling proteins, including Cav1.2, NCX, RyR2, SERCA2a, phospholamban (PLB), calsequestrin (CSQ), and junctophilin-2 (JP2), was comparable between WT and *Tric-a^−/−^* hearts under baseline condition (Figure 1C). TRIC-B protein abundance was also unchanged, indicating that TRIC isoforms do not compensate for each other at the expression level in adult myocardium.

Because functional alterations were observed in the absence of changes in protein abundance, we next examined phosphorylation states of key Ca^2+^-regulatory proteins. Using phospho-specific antibodies, we identified a striking increase in RyR2 phosphorylation at serine 2808 (S2808) in *Tric-a^−/−^* hearts [42,43], whereas phosphorylation at serine 2814 (S2814), a CaMKII target site, remained unchanged [43,44,45,46]. Parallel analysis revealed a significant elevation in PLB phosphorylation at serine 16 (S16) [47,48,49], the PKA-dependent site, but no detectable change at threonine 17 (T17) [50], the CaMKII-dependent site (Figure 1C,D).

These phosphorylation patterns are consistent with increased basal phosphorylation at PKA-preferred sites in TRIC-A-deficient hearts. Enhanced RyR2-S2808 and PLB-S16 phosphorylation would be expected to promote SR Ca^2+^ release and accelerate Ca^2+^ reuptake, respectively, and may represent compensatory adjustments to maintain contractility despite impaired local RyR2 activation in the absence of TRIC-A.

### 3.2. Acute β-Adrenergic Stimulation Provokes Mitochondrial Injury in TRIC-A Deficient Hearts

The imbalance between local and global Ca^2+^ release observed in *Tric-a^−/−^* cardiomyocytes raised the possibility that mitochondria, major Ca^2+^ buffers and metabolic regulators, may be particularly vulnerable to overload during β-adrenergic stimulation. To evaluate this, we examined cardiac ultrastructure following 24 h of continuous ISO infusion.

**Figure 2:**
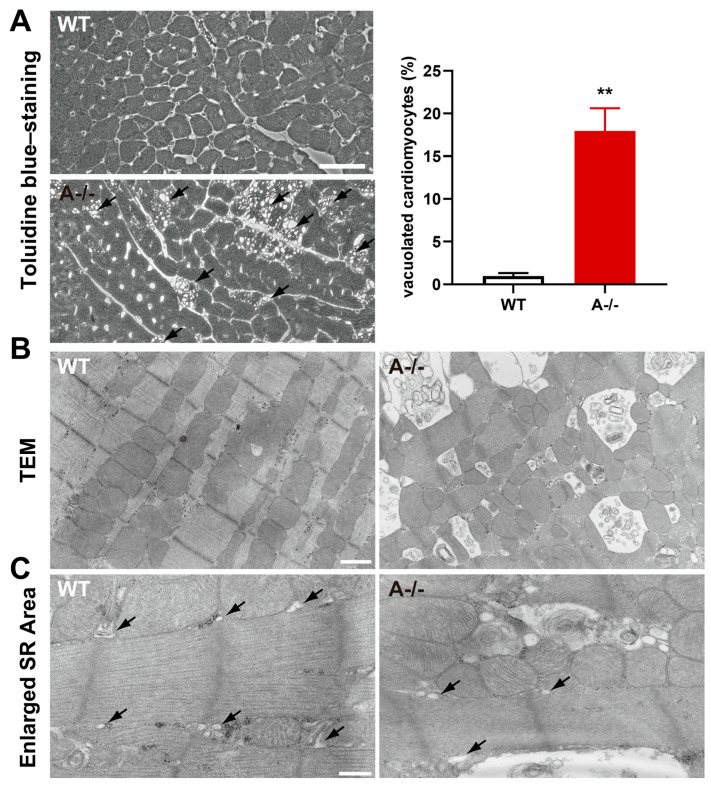
**Mitochondrial abnormalities in ISO-treated *Tric-a^−/−^* hearts.** (**A**) Toluidine blue-stained ventricular sections from WT and *Tric-a^−/−^* hearts after 24 h ISO treatment, showing vacuolated cardiomyocytes. Arrows indicate vacuole-positive cells (*n* = 6). Mann–Whitney test ** *p* < 0.01. (**B**) Transmission electron micrographs of WT and *Tric-a^−/−^* cardiomyocytes. Abnormal mitochondria in *Tric-a^−/−^* cardiomyocytes showing swelling and disrupted cristae. (**C**) Enlarged cardiomyocyte area. Arrows indicate SR. Scale bars: 50 μm (**A**), 1 μm (**B**), and 400 nm (**C**).

Light microscopy revealed prominent vacuolization in *Tric-a^−/−^* hearts, whereas WT myocardium displayed no overt vacuoles (Figure 2A). Quantitative analysis demonstrated that 17.9 ± 2.7% of *Tric-a^−/−^* cardiomyocytes contained visible vacuoles, indicating a widespread structural response to acute adrenergic stress (Figure 2A). To determine the origin of these vacuoles, we performed transmission electron microscopy.

Electron micrographs showed numerous low-density vacuoles dispersed throughout *Tric-a^−/−^* cardiomyocytes (Figure 2C). Importantly, the sarcoplasmic reticulum appeared structurally intact in TRIC-A-deficient cells (Figure 2C), consistent with preserved SR membrane organization. In contrast, striking mitochondrial abnormalities were evident in *Tric-a^−/−^* hearts (Figure 2B), including swelling, disrupted cristae, and increased electron-lucent regions, hallmarks of mitochondrial permeability transition and Ca^2+^-induced degeneration.

These observations indicate that mitochondrial dysfunction, rather than SR fragmentation, underlies the vacuolization phenotype in TRIC-A-deficient myocardium. The data support a model in which dysregulated SR Ca^2+^ release in the absence of TRIC-A directly destabilizes mitochondrial integrity, rendering cardiomyocytes susceptible to acute β-adrenergic stress. Such vulnerability aligns with the known requirement for precise SR–mitochondrial Ca^2+^ coupling to maintain energetic balance and protect against Ca^2+^ overload-induced injury.

### 3.3. Cardiomyocyte Death with Loss of Membrane Integrity Follows Mitochondrial Injury

**Figure 3:**
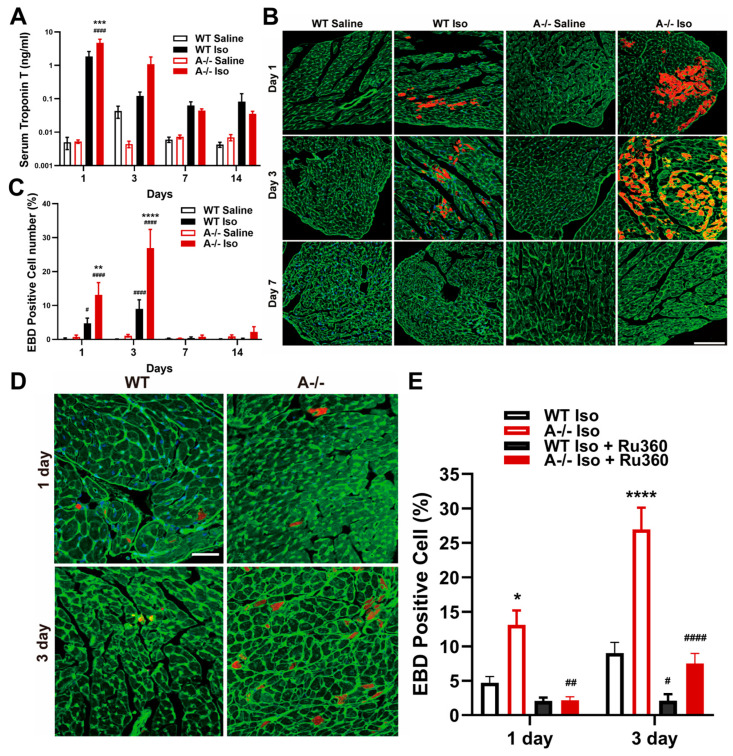
**Cardiomyocyte injury and ISO-induced cell death with rescue by MCU inhibition.** (**A**) Serum cardiac troponin T levels over time in WT and *Tric-a^−/−^* mice after saline or ISO (*n* = 3–5). Three-way ANOVA showed no main effect of genotype; Post Hoc Tukey’s test indicates a difference at Day 1 **** *p* < 0.0001 Vs. WT #### *p* < 0.0001 Vs. Saline. (**B**) Evans blue dye (EBD)-positive cardiomyocytes over time in WT and *Tric-a^−/−^* hearts after saline or ISO. Scale bars = 100 µm. (**C**) ISO-induced cell death in isolated cardiomyocytes (*n* = 3). Three-way ANOVA revealed significant main effects and a genotype × treatment × day interaction, all *p* < 0.0001. (**D**) Representative images of EBD-stained cardiomyocytes after ISO with or without Ru360, a mitochondrial Ca^2+^ uniporter inhibitor. Scale bars = 500 µm. (**E**) Quantification of EBD-positive cardiomyocytes showing significant rescue by Ru360 in *Tric-a^−/−^* hearts (*n* = 3). Three-way ANOVA revealed significant main effects of genotype, treatment, and day (all *p* < 0.0001), with significant treatment × genotype (*p* < 0.001), and day × genotype (*p* < 0.01) interactions. Symbols indicate Tukey’s multiple comparisons *, **, ***, **** *p* < 0.05, 0.01, 0.001, 0.0001 Vs. WT and #, ##, #### *p* < 0.05, 0.01, 0.0001 Vs. Saline.

Given the profound mitochondrial abnormalities observed in *Tric-a^−/−^* hearts under acute β-adrenergic stimulation, we next asked whether these structural defects were accompanied by cardiomyocyte death. Circulating cardiac troponin T, a sensitive marker released upon sarcolemmal disruption, was markedly elevated in *Tric-a^−/−^* mice after 24 h of ISO infusion compared with WT controls (Figure 3A). This systemic increase in troponin T suggests widespread loss of membrane integrity in the absence of TRIC-A.

To directly evaluate membrane permeability at the cellular level, we used Evans blue dye (EBD), which selectively accumulates in cardiomyocytes with irreversible sarcolemmal damage. *Tric-a^−/−^* hearts demonstrated a robust increase in EBD-positive cells as early as day 1 following ISO infusion, with the proportion of injured cardiomyocytes peaking at day 3 before resolving by day 7 (Figure 3B,C). WT hearts displayed only minimal EBD incorporation across all time points, indicating that β-adrenergic stimulation alone is insufficient to provoke comparable membrane damage.

To determine whether mitochondrial Ca^2+^ overload was a mechanistic driver of this injury, we treated mice with Ru360, a selective inhibitor of the mitochondrial Ca^2+^ uniporter (MCU) [51,52,53,54]. Ru360 treatment markedly reduced the number of EBD-positive cardiomyocytes in *Tric-a^−/−^* hearts (Figure 3D,E), demonstrating that excessive mitochondrial Ca^2+^ uptake is required for the membrane-disruptive injury phenotype. This rescue effect directly links dysregulated SR–mitochondrial Ca^2+^ transfer to cardiomyocyte death under β-adrenergic stress.

### 3.4. Sustained β-Adrenergic Stimulation Drives Fibrotic Remodeling in TRIC-A Deficient Hearts

**Figure 4:**
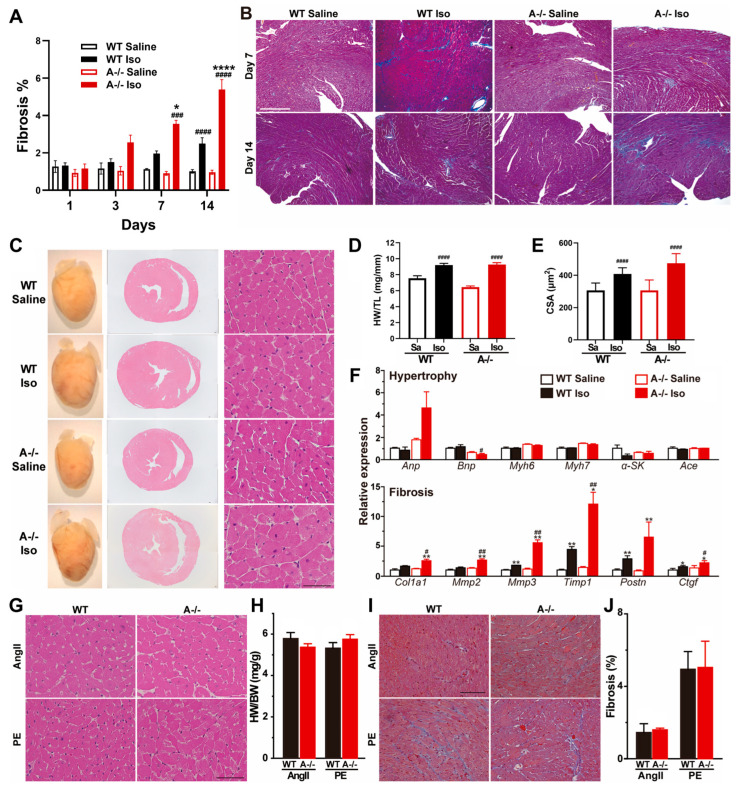
**ISO-induced fibrosis and remodeling specificity to β-adrenergic stress.** (**A**) Time course of cardiac fibrosis quantified in WT and *Tric-a^−/−^* hearts after saline or ISO (*n* = 3–18). Three-way ANOVA revealed significant main effects of genotype (*p* < 0.01), treatment (*p* < 0.0001), and day (*p* < 0.0001), with significant treatment × day (*p* < 0.0001), treatment × genotype (*p* < 0.001), and day × genotype (*p* < 0.05) interactions. (**B**) Representative Masson’s trichrome-stained ventricular sections after 14 days saline or ISO. (**C**) Gross heart images and H&E-stained cross-sections after 14 days saline or ISO. (**D**) Heart weight-to-tibia length ratio (*n* = 16–19). Two-way ANOVA revealed significant main effects of genotype (*p* < 0.05) and treatment (*p* < 0.0001), as well as a significant genotype × treatment interaction (*p* < 0.05). (**E**) Cardiomyocyte cross-sectional area (*n* = 15–16). Two-way ANOVA revealed significant main effects of genotype (*p* < 0.05) and treatment (*p* < 0.0001), as well as a significant genotype × treatment interaction (*p* < 0.05). (**F**) Relative mRNA expression of hypertrophy markers (upper) and fibrosis markers (lower) in WT and *Tric-a^−/−^* hearts with or without ISO (*n* = 4). Two-way ANOVA revealed significant main effects of genotype for all fibrosis markers. (**G**) Representative HE-stained sections after 14 days angiotensin II (AngII) or phenylephrine (PE) treatment. (**H**) Heart weight-to-body weight ratio after AngII or PE (*n* = 6). (**I**), Masson’s trichrome-stained ventricular sections after 14 days AngII or PE. (**J**) Quantification of fibrotic area after AngII or PE (*n* = 6–7). Two-way ANOVA found no main effects and no interaction (**H**,**J**). Symbols indicate Tukey’s multiple comparisons *, **, **** *p* < 0.05, 0.01, 0.0001 Vs. WT and #, ##, ###, #### *p* < 0.05, 0.01, 0.001, 0.0001 Vs. Saline.

Because cardiomyocyte death often initiates maladaptive tissue remodeling, we next examined whether prolonged β-adrenergic stimulation leads to chronic pathological changes in *Tric-a^−/−^* hearts. Histological assessment after 14 days of continuous ISO infusion revealed extensive interstitial and perivascular fibrosis throughout *Tric-a^−/−^* ventricles, whereas WT hearts displayed only mild fibrotic deposition (Figure 4A,B). Fibrosis was particularly pronounced within the left ventricular free wall, aligning with regions that exhibited acute injury and membrane disruption in earlier phases of the model.

Consistent with these structural findings, *Tric-a^−/−^* hearts showed marked transcriptional activation of fibrosis-associated genes, including *Col1a1*, *Mmp3*, *Timp1*, and *Ctgf*, all of which were significantly upregulated compared with WT (Figure 4F). These molecular signatures confirm a robust profibrotic response downstream of sustained catecholaminergic stress.

Despite the prominent fibrotic remodeling, indices of hypertrophic growth remained largely unchanged between genotypes. Heart weight-to-tibia length ratios, cardiomyocyte cross-sectional area, and classical hypertrophy marker expression were comparable in WT and *Tric-a^−/−^* hearts following ISO treatment (Figure 4D–F). We observed that following ISO treatment, *Bnp* expression appeared to be reduced in *Tric-a^−/−^* hearts, whereas *Anp* expression is increased. The divergent regulation of these canonical hypertrophic markers precludes a definitive conclusion regarding the role of TRIC-A in modulating the cardiac hypertrophic response under β-adrenergic stress.

To determine whether this selective remodeling phenotype was unique to β-adrenergic stimulation, we subjected mice to angiotensin II (AngII) or phenylephrine (PE), two established hypertrophic stimuli. Under both conditions, WT and *Tric-a^−/−^* hearts developed similar degrees of hypertrophy and fibrosis, with no genotype-dependent differences (Figure 4G–J). These results indicate that TRIC-A deficiency does not globally sensitize the heart to hypertrophic or fibrotic remodeling, but rather exhibits specificity to β-adrenergic pathways.

### 3.5. Cardiac Fibroblasts Lack TRIC-A Expression and Exhibit Normal Proliferation and Ca^2+^ Handling

**Figure 5:**
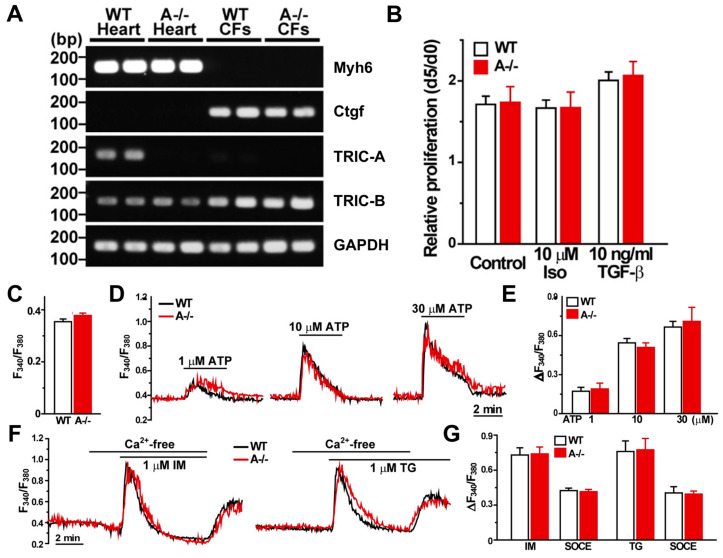
**Cardiac fibroblasts lack TRIC-A expression and exhibit normal proliferation and Ca^2+^ handling.** (**A**) RT-PCR analysis of *Myh6* (cardiomyocyte marker), *Ctgf* (cardiac fibroblast marker), *Tric-a*, *Tric-b*, and *Gapdh* (internal control). *Tric-a* mRNA was readily detected in cardiomyocytes but was absent in cardiac fibroblasts (CFs), whereas *Tric-b* was expressed in both populations. (**B**) Quantification of fibroblast proliferation shows no difference between WT and *Tric-a^−/−^* CFs (*n* = 4). C-G, Fura-2 Ca^2+^ imaging in CFs isolated from WT and *Tric-a^−/−^* mice. (**C**) Resting cytosolic Ca^2+^ levels under basal conditions. (**D**) Representative traces of ATP-induced Ca^2+^ transients in response to increasing ATP concentrations. (**E**) Averaged ATP-evoked Ca^2+^ responses demonstrating comparable purinergic signaling between genotypes (*n* = 5–6). (**F**), Representative traces showing ionomycin (IM)- and thapsigargin (TG)-induced Ca^2+^ release, followed by store-operated Ca^2+^ entry (SOCE). (**G**) Quantification of IM- and TG-evoked Ca^2+^ responses and subsequent SOCE (*n* = 4–6). Overall, Ca^2+^ signaling properties, including basal Ca^2+^ levels, ATP responsiveness, SR store content, and SOCE, were indistinguishable between WT and *Tric-a^−/−^* fibroblasts. Two-way ANOVA revealed no significant main effects of genotype or treatment and no significant interactions for any measured parameter. Original electrophoresis images can be found in Supplementary Materials.

Given the pronounced fibrosis observed in *Tric-a^−/−^* hearts following β-adrenergic stimulation, we next examined whether cardiac fibroblasts (CFs) themselves contribute to this exaggerated remodeling response. Type I collagen–positive, α-actinin–negative CFs were isolated and cultured from WT and *Tric-a^−/−^* hearts following the protocol described in [41] (Figure 5). As expected, CFs expressed high levels of the fibroblast marker *Ctgf*, whereas the cardiomyocyte marker *Myh6* (Myhc-α) was absent (Figure 5A). RT-PCR confirmed that *Tric-a* mRNA was undetectable in WT CFs, while *Tric-a* was readily detected in cardiomyocytes, indicating that fibroblasts do not normally express this channel and are unlikely to be directly impacted by its deletion (Figure 5A).

To assess whether TRIC-A loss alters fibroblast behavior, we first evaluated cell proliferation. Proliferation rates were indistinguishable between WT and *Tric-a^−/−^* CFs, and exposure to either 10 μM isoproterenol or 10 ng/mL TGF-β did not modify proliferation in either genotype (Figure 5B).

We next performed Fura-2 Ca^2+^ imaging to evaluate fibroblast Ca^2+^ signaling. Baseline cytosolic Ca^2+^ levels were comparable between WT and *Tric-a^−/−^* CFs (Figure 5C). ATP-evoked Ca^2+^ transients showed similar amplitude and kinetics in both groups (Figure 5D,E), indicating normal purinergic signaling. Ionomycin (IM)- and thapsigargin (TG)-evoked SR Ca^2+^ release, as well as subsequent store-operated Ca^2+^ entry (SOCE), were likewise unchanged by TRIC-A deletion (Figure 5F,G). Overall, basal Ca^2+^ levels, ATP responsiveness, intracellular Ca^2+^ store content, and SOCE were equivalent between genotypes.

Together, these results demonstrate that cardiac fibroblasts neither express TRIC-A nor exhibit genotype-dependent differences in growth, Ca^2+^ handling, or profibrotic activation. The absence of intrinsic fibroblast abnormalities strongly supports the conclusion that the exaggerated fibrosis observed in *Tric-a^−/−^* hearts is a secondary response to cardiomyocyte injury and necrosis rather than a fibroblast-driven primary defect.

## 4. Discussion

In this study, we identify TRIC-A as a critical stabilizer of SR Ca^2+^ release and cardiac stress resilience. Although TRIC-A has long been implicated in counter-ion conductance and RyR2 regulation, its physiological significance in the intact adult heart has remained incompletely understood. Here, we show that loss of TRIC-A disrupts the balance between local and global Ca^2+^ release, renders mitochondria vulnerable to Ca^2+^ overload during acute β-adrenergic stimulation, precipitates necrosis-like cardiomyocyte death, and ultimately drives a secondary fibrotic remodeling program. These findings establish TRIC-A as a central gatekeeper that links SR Ca^2+^ homeostasis to mitochondrial integrity and tissue-level remodeling.

### 4.1. TRIC-A as a Dual Regulator of SR Ca^2+^ Release

Our results confirm and extend earlier observations that TRIC-A deficiency alters RyR2-mediated Ca^2+^ signaling. *Tric-a^−/−^* myocytes exhibit reduced spontaneous Ca^2+^ spark activity but elevated global caffeine-evoked release [20,39], and increased Ca^2+^ transient amplitude following isoproterenol stimulation, hallmarks of SR Ca^2+^ overload. These phenomena are consistent with the established concept that TRIC-A provides counter-ion flux necessary to dissipate electrochemical gradients that develop during Ca^2+^ efflux through RyR2 [13]. In the absence of sufficient counter-current, local release is dampened while overall SR Ca^2+^ content rises.

At the molecular level, TRIC-A has been shown to interact with RyR2 through its C-terminal tail and enhance RyR2 open probability [19]. In agreement, *Tric-a^−/−^* hearts display phosphorylation changes in RyR2 (S2808) and PLB (S16) that are characteristic of compensatory β-adrenergic signaling. These modifications likely reflect attempts to augment RyR2 opening and SERCA activity in the face of impaired local Ca^2+^ release. Thus, TRIC-A appears to serve dual complementary functions: (1) providing the ionic conditions necessary for efficient SR Ca^2+^ release, and (2) directly modulating RyR2 gating to tune local Ca^2+^ signaling.

### 4.2. β-Adrenergic Stimulation Exposes a Latent Vulnerability in TRIC-A Deficiency

A major finding of this study is that TRIC-A deficiency renders cardiomyocytes highly susceptible to acute β-adrenergic stress. Isoproterenol stimulation, which shifts RyR2 gating and increases SR Ca^2+^ turnover, precipitated mitochondrial swelling, cristae disruption, and electron-lucent vacuolization in *Tric-a^−/−^* hearts, features classic for Ca^2+^-dependent mitochondrial permeability transition. Importantly, SR ultrastructure remained intact, suggesting that mitochondrial destabilization is not secondary to structural collapse of Ca^2+^ stores but arises directly from dysregulated SR-to-mitochondrial Ca^2+^ transfer.

The fact that Ru360 treatment attenuated EBD uptake in *Tric-a^−/−^* hearts firmly implicates mitochondrial Ca^2+^ overload as the proximate trigger for cardiomyocyte death. These results also highlight that TRIC-A, though localized to the SR, indirectly governs mitochondrial integrity by ensuring appropriately calibrated Ca^2+^ release during β-adrenergic activation. When this regulation is lost, heightened SR Ca^2+^ load combined with β-adrenergic augmentation overwhelms mitochondrial buffering capacity, leading to necrotic membrane rupture rather than apoptotic signaling. These data position TRIC-A as a critical stabilizer of Ca^2+^ flux between the SR and mitochondria, safeguarding cardiomyocyte viability during acute neurohumoral stress.

### 4.3. Necrosis-Driven Fibrosis as the Dominant Remodeling Pathway

Because cardiomyocyte necrosis is a strong stimulus for fibrotic repair, we examined whether TRIC-A deficiency alters chronic remodeling outcomes. Sustained β-adrenergic stimulation induced extensive interstitial and perivascular fibrosis in *Tric-a^−/−^* hearts, accompanied by robust upregulation of fibrogenic genes (*Col1a1*, *Mmp3*, *Timp1*, *Ctgf*). Notably, hypertrophic remodeling was not exaggerated, and *Tric-a^−/−^* hearts responded normally to AngII- or PE-induced hypertrophy.

These findings underscore that fibrosis in TRIC-A deficiency is injury-driven rather than hypertrophy-driven. This response is specific to β-adrenergic stress, consistent with the unique ability of catecholamines to destabilize Ca^2+^ handling when SR regulatory mechanisms fail. Importantly, fibroblasts themselves did not express *Tric-a* and showed no genotype-dependent differences in growth, Ca^2+^ signaling, or TGF-β-induced activation. This rules out a fibroblast-intrinsic defect and strengthens the conclusion that fibrosis is a secondary consequence of cardiomyocyte necrosis initiated by mitochondrial Ca^2+^ overload.

### 4.4. Implications for Cardiac Physiology and Disease

Our findings provide mechanistic insight into how intracellular ionic countercurrents maintain cardiac resilience during stress. While RyR2 has dominated attention as the central SR Ca^2+^ release channel, these data highlight the importance of ancillary proteins, such as TRIC-A, that support the electrochemical environment needed for stable release. Loss of TRIC-A reveals an underappreciated vulnerability: RyR2-mediated Ca^2+^ release becomes uncoupled from its mitochondrial buffering partner, predisposing the heart to catastrophic failure when catecholamine levels rise. The combination of acute cardiomyocyte necrosis and mitochondrial Ca^2+^-dependent membrane rupture provides a mechanistic basis for the selective fibrotic phenotype, distinguishing β-adrenergic-induced remodeling from classic hypertrophic responses. These insights may have broader implications for conditions characterized by heightened adrenergic drive and mitochondrial dysfunction, including pressure overload, arrhythmogenic disorders, and adrenergic crisis states. They also suggest that modulation of TRIC-A function, or restoration of balanced SR counter-ion flux, could represent a therapeutic strategy to prevent Ca^2+^ overload-induced myocardial injury.

### 4.5. Limitations and Future Directions

Although this study establishes TRIC-A as a key regulator of SR–mitochondrial Ca^2+^ coupling and β-adrenergic stress resilience, several limitations warrant further investigation. First, experiments were performed in male mice. Therefore, future studies will be required to determine whether the observed phenotypes are conserved in females.

Second, our work focuses primarily on global *Tric-a* knockout mice, which effectively model loss of TRIC-A function but do not distinguish cardiomyocyte-specific versus systemic contributions. While fibroblasts lack *Tric-a* expression and show no genotype-dependent phenotype, future studies using cardiomyocyte-specific and inducible *Tric-a* deletion models will be important to definitively establish cell-autonomous roles and to determine whether the susceptibility to adrenergic injury arises during development or in adulthood. Moreover, whether partial TRIC-A loss, akin to human genetic variation or acquired downregulation during heart disease, similarly compromises mitochondrial resilience remains an open question.

Third, we assessed RyR2 and phospholamban phosphorylation primarily under baseline conditions, so β-adrenergic stress–dependent regulation of these phosphorylation endpoints was not directly examined. Future studies should quantify phosphorylation responses to acute ISO stimulation and other β-adrenergic challenges to determine whether TRIC-A deficiency alters stimulus-dependent signaling dynamics.

Fourth, while our results clearly differentiate β-adrenergic-induced fibrosis from AngII- or PE-driven hypertrophy, the molecular basis for this stimulus specificity is not yet understood. Because β-adrenergic signaling uniquely enhances RyR2 activity and accelerates SR Ca^2+^ turnover, future work should investigate whether TRIC-A’s regulatory influence becomes particularly critical under conditions of heightened Ca^2+^ flux, arrhythmogenic stress, or catecholamine-driven cardiomyopathies such as Takotsubo syndrome.

Fifth, the translational implications of TRIC-A dysfunction merit further exploration. Human genetic studies have identified variants in the *TMEM38A* locus [15,28], but the mechanistic significance of these variants remains poorly characterized. Determining whether TMEM38A dysregulation contributes to human cardiomyopathy or stress-induced cardiac injury would provide an important bridge from basic Ca^2+^-handling biology to clinical application. In parallel, therapeutic strategies aimed at modulating SR counter-ion flux, stabilizing RyR2, or limiting mitochondrial Ca^2+^ overload may represent new avenues to protect the heart from catecholamine-induced injury.

## 5. Conclusions

Together, our findings establish TRIC-A as an essential regulator of SR Ca^2+^ release, mitochondrial integrity, and cardiac stress tolerance. By controlling both the ionic microenvironment and the functional behavior of RyR2, TRIC-A prevents excessive SR Ca^2+^ loading and protects mitochondria from Ca^2+^-dependent injury during β-adrenergic activation. Its absence shifts the cardiac injury response toward necrosis and fibrosis, unveiling a critical molecular axis that integrates Ca^2+^ handling with tissue remodeling. These results advance our understanding of intracellular Ca^2+^ homeostasis and identify TRIC-A as a pivotal determinant of cardiac resilience under neurohumoral stress.

## Data Availability

The original contributions presented in this study are included in the article/Supplementary Material. Further inquiries can be directed to the corresponding author.

## Supplementary Materials

The following supporting information can be downloaded at: https://www.mdpi.com/article/10.3390/biom16020181/s1, Table S1: RT-qPCR primer sequences. Original western blots images and electrophoresis images also in Supplementary Materials.

## Abbreviations

The following abbreviations are used in this manuscript:

AngII	Angiotensin II
ATP	Adenosine triphosphate
BDM	2,3-Butanedione monoxime
CaMKII	Ca^2+^/calmodulin-dependent protein kinase II
CFs	Cardiac fibroblasts
CSQ/CSQs	Calsequestrin
EBD	Evans blue dye
ECLIA	Electrochemiluminescence immunoassay
ER	Endoplasmic reticulum
HE	Hematoxylin and eosin
HEPES	4-(2-hydroxyethyl)-1-piperazineethanesulfonic acid
IM	Ionomycin
ISO	Isoproterenol
JP2	Junctophilin-2
MCU	Mitochondrial Ca^2+^ uniporter
MT	Masson trichrome
NCX/NCX1	Na^+^/Ca^2+^ exchanger (isoform 1)
PBS	Phosphate-buffered saline
PKA	Protein kinase A
PLB	Phospholamban
PVDF	Polyvinylidene fluoride
RT-PCR	Reverse transcription polymerase chain reaction
RyR2	Ryanodine receptor 2
SOCE	Store-operated Ca^2+^ entry
SOICR	Store-overload-induced Ca^2+^ release
SR	Sarcoplasmic reticulum
TEM	Transmission electron microscopy
TG	Thapsigargin
TMEM38A	Transmembrane protein 38A
TMEM38B	Transmembrane protein 38B
TRIC	Trimeric intracellular cation channel
TRIC-A	Trimeric intracellular cation channel A (TMEM38A)
TRIC-B	Trimeric intracellular cation channel B (TMEM38B)
WT	Wild type
