# Supplementary material for: TRIC-A Loss Sensitizes the Heart to β-Adrenergic Stress and Drives Cardiomyocyte Death and Fibrosis
Source: Biomolecules. 2026 Jan 23;16(2):181. doi: 10.3390/biom16020181 (PMC12937667; doi:10.3390/biom16020181)

Supplemental information

Supplemental Table S1. RT-qPCR primer sequences

Target gene	Forward	Reverse
Anp	CCTGTGTACAGTGCGGTGTC	TCTCAGAGGTGGGTTGACCT
Bnp	AGGTGCTGTCCCAGATGATT	CCTTGGTCCTTCAAGAGCTG
Myh6	CGCCTATGAGGAGTCTCTGG	TTCTCCACCTCCAGCTGTTT
Myh7	TCCCAGCTCCAGACAGAAGT	TGCAAGTCCTTGATGGTCTG
α-SK	AAGTGCGACATCGACATCAG	CCACCGATCCACACTGAGTA
Ace	CAGTGTCTACCCCCAAGCAT	TTCCATCAAAGACCCTCCAG
Col1a1	GAGCGGAGAGTACTGGATCG	GCTTCTTTTCCTTGGGGTTC
Mmp2	TGGGGGAGATTCTCACTTTG	CATCACTGCGACCAGTGTCT
Mmp3	CAGACTTGTCCCGTTTCCAT	GGTGCTGACTGCATCAAAGA
Timp1	ATTCAAGGCTGTGGGAAATG	CTCAGAGTACGCCAGGGAAC
Postn	AACCAAGGACCTGAAACACG	GTGTCAGGACACGGTCAATG
Ctgf	AGCAGCTGGGAGAACTGTGT	GCTGCTTTGGAAGGACTCAC
TRIC-A	CTTCCCTGTTTTCGACCTCA	GCCAGGATGTAACTCCCAAA
TRIC-B	TGAAGATGTCCTTCCCTTGC	AGGGTGTCCTCAAAGGGAGT
GAPDH	GGCATTGCTCTCAATGACAA	TGTGAGGGAGATGCTCAGTG

Uncropped Western blots images

Figure 1. C – $Ca_v1.2$

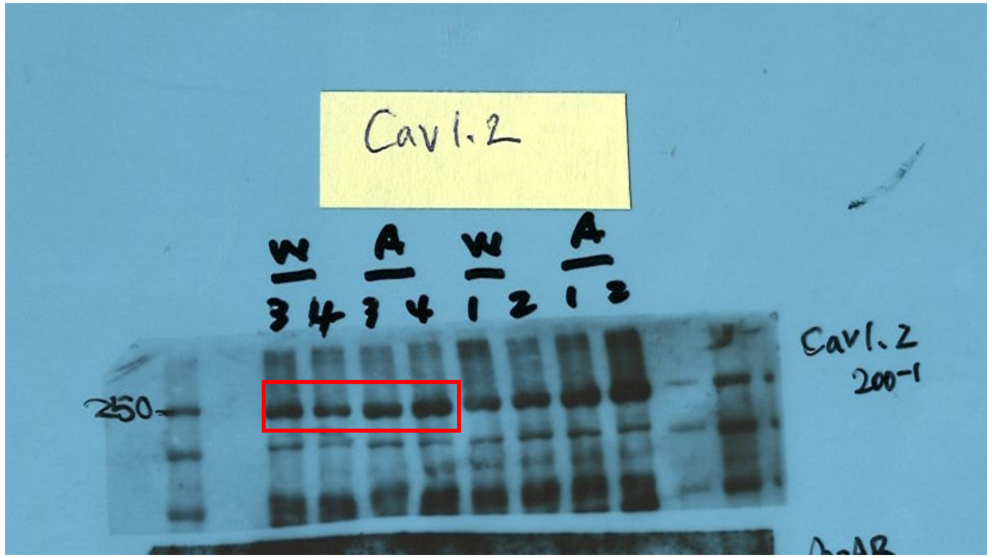

Figure 1. C – pRyR2 (Ser2808)

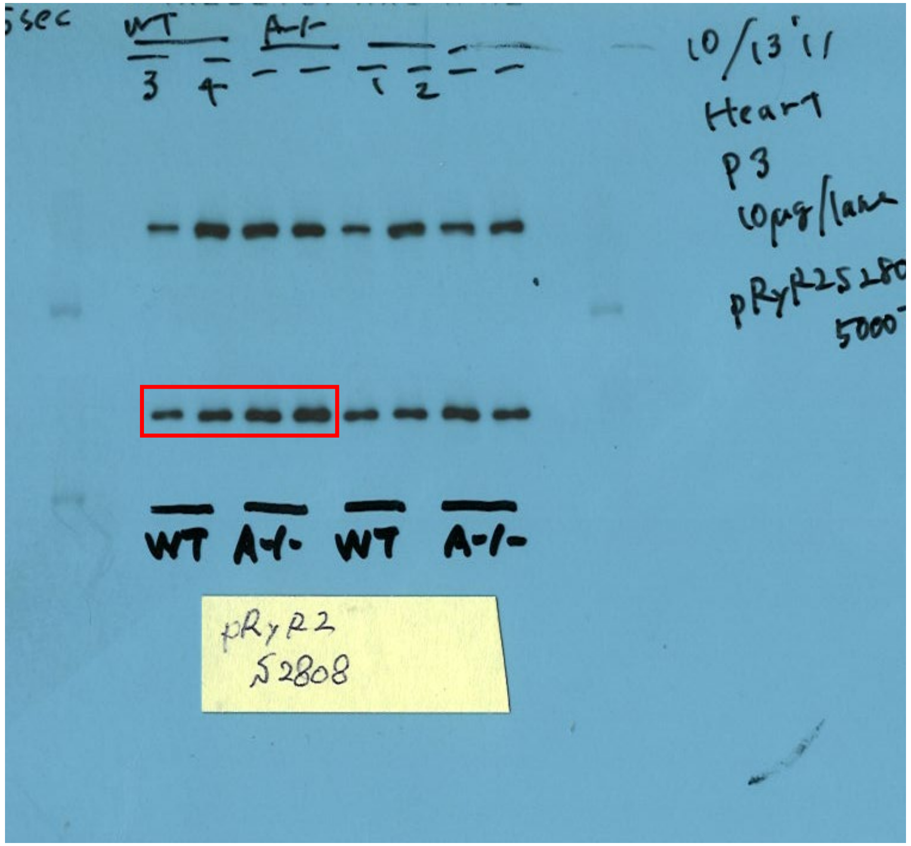

Figure 1. C – pRyR2 (Ser2814)

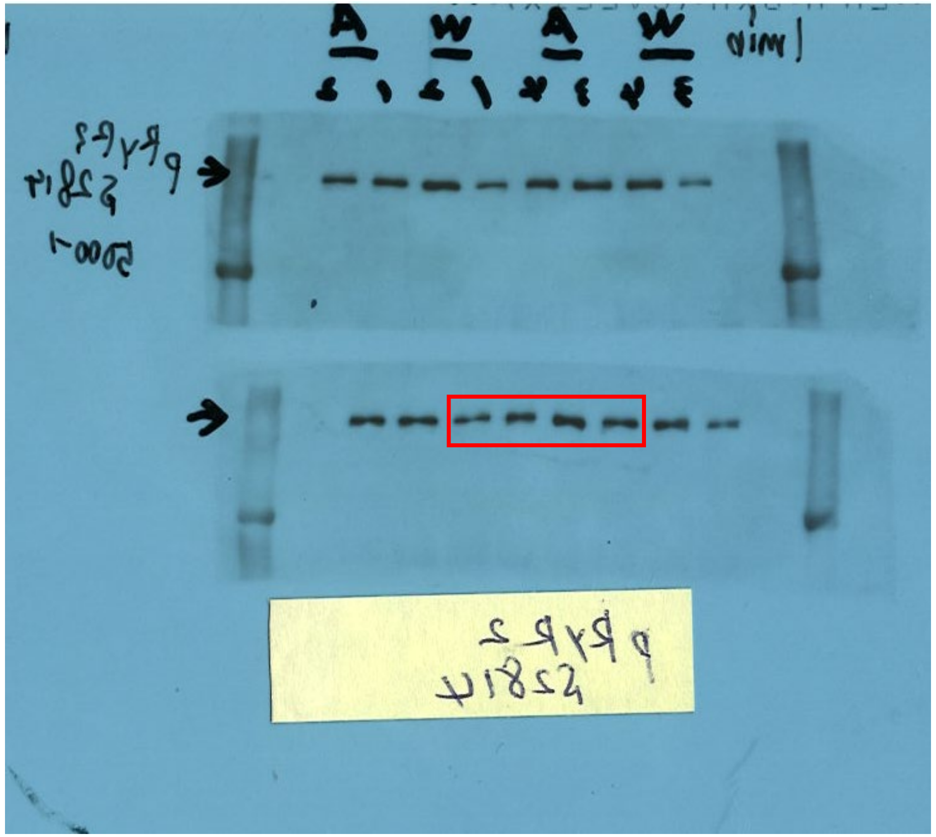

Figure 1. C – RyR2

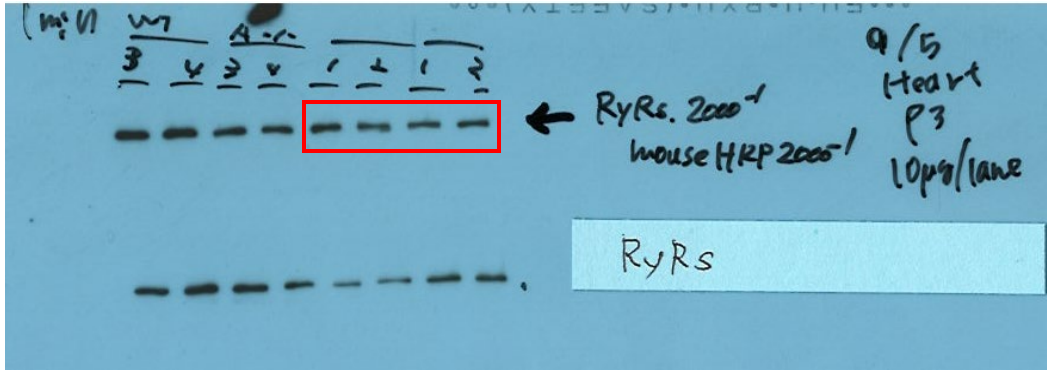

Figure 1. C – IP_3R2

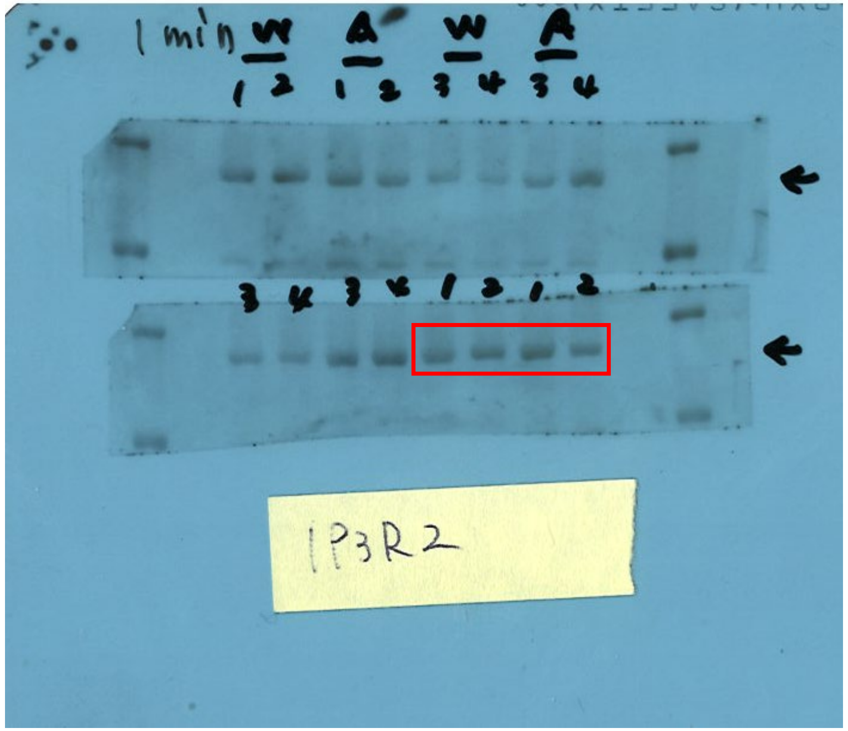

Figure 1. C – NCX1

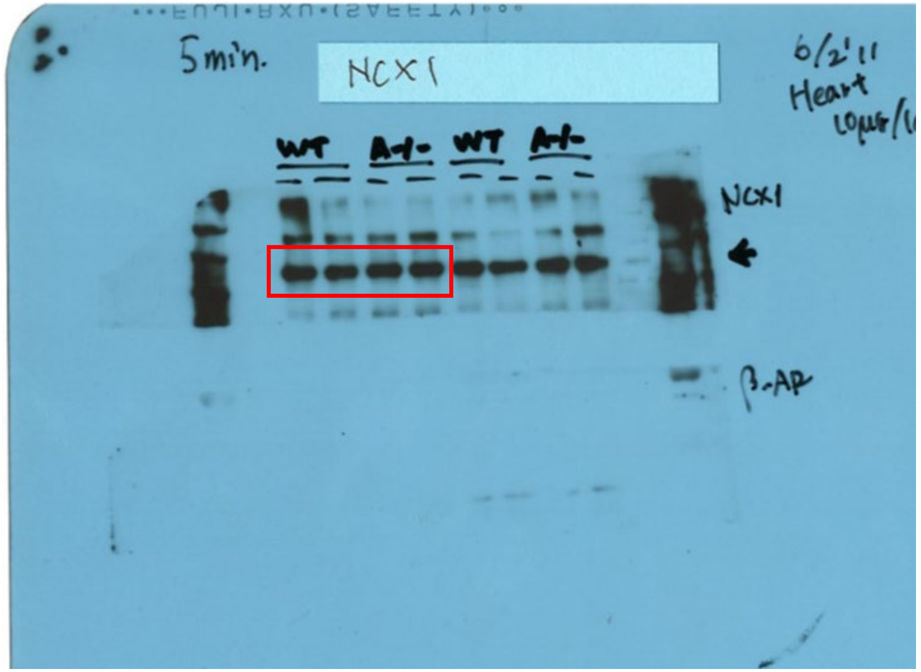

Figure 1. C – pPLB (Ser16)

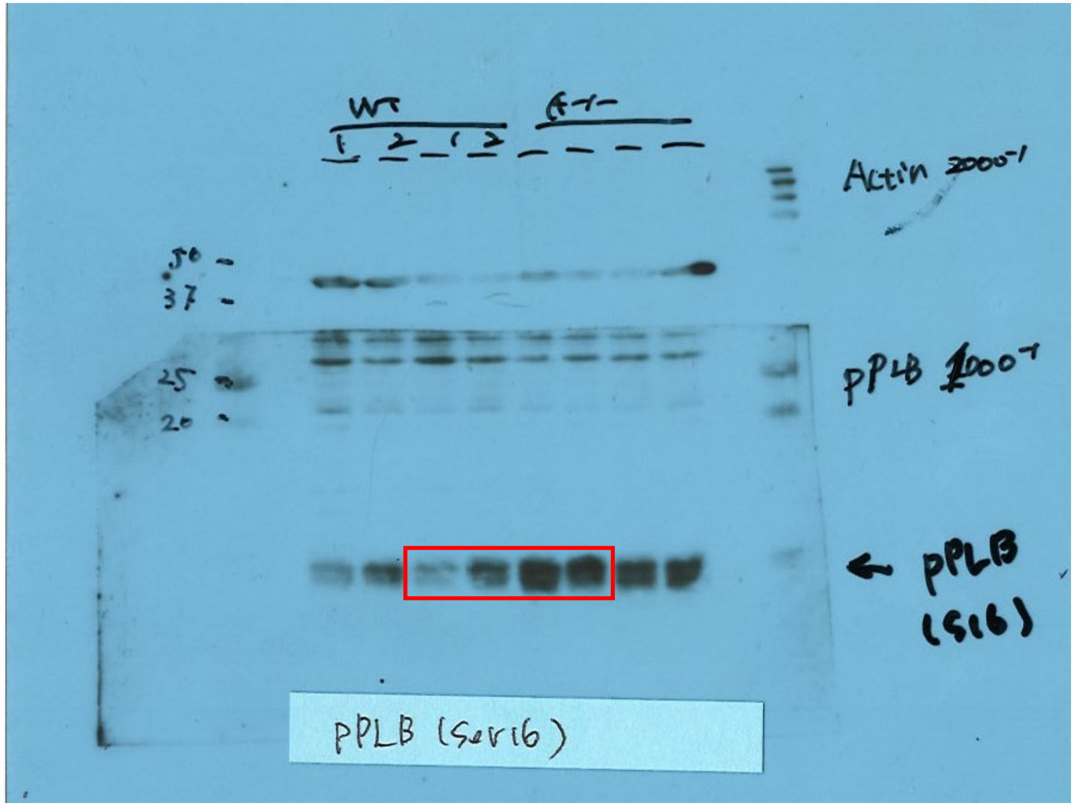

Figure 1. C – pPLB (Thr17)

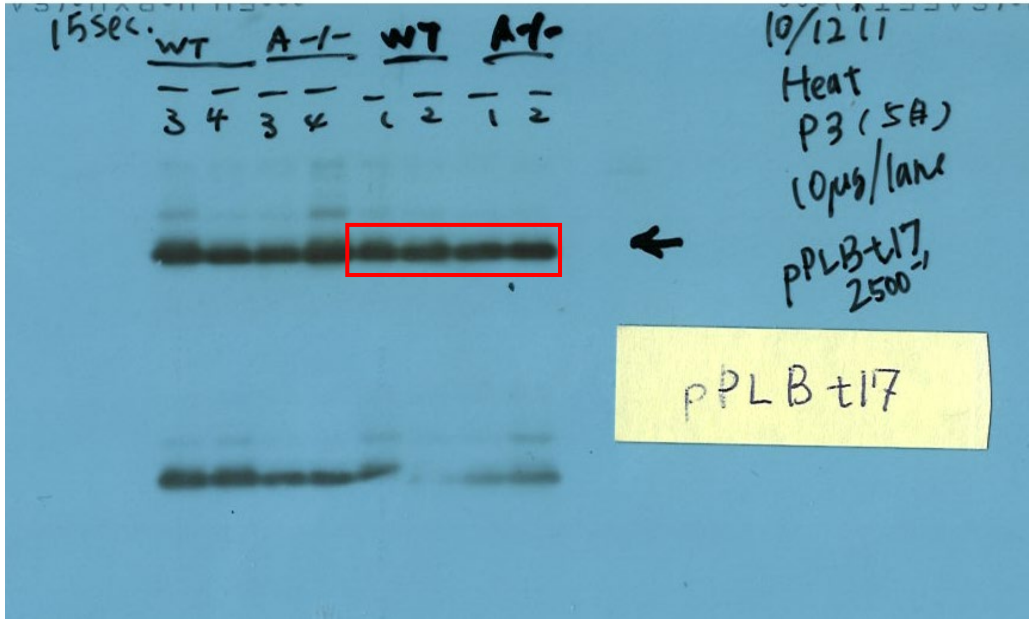

Figure 1. C – PLB

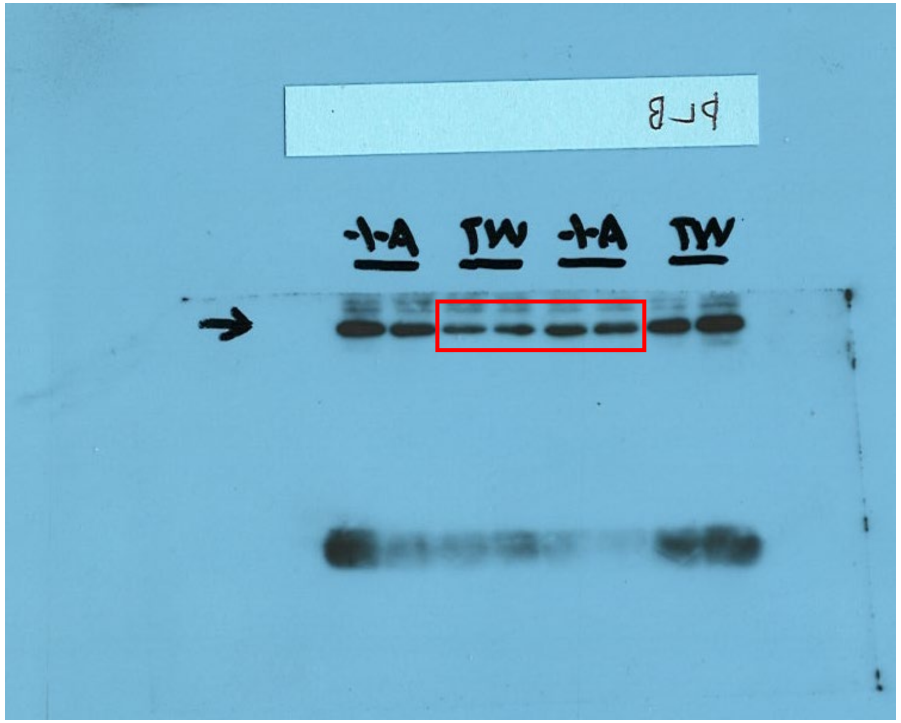

Figure 1. C – SERCA2

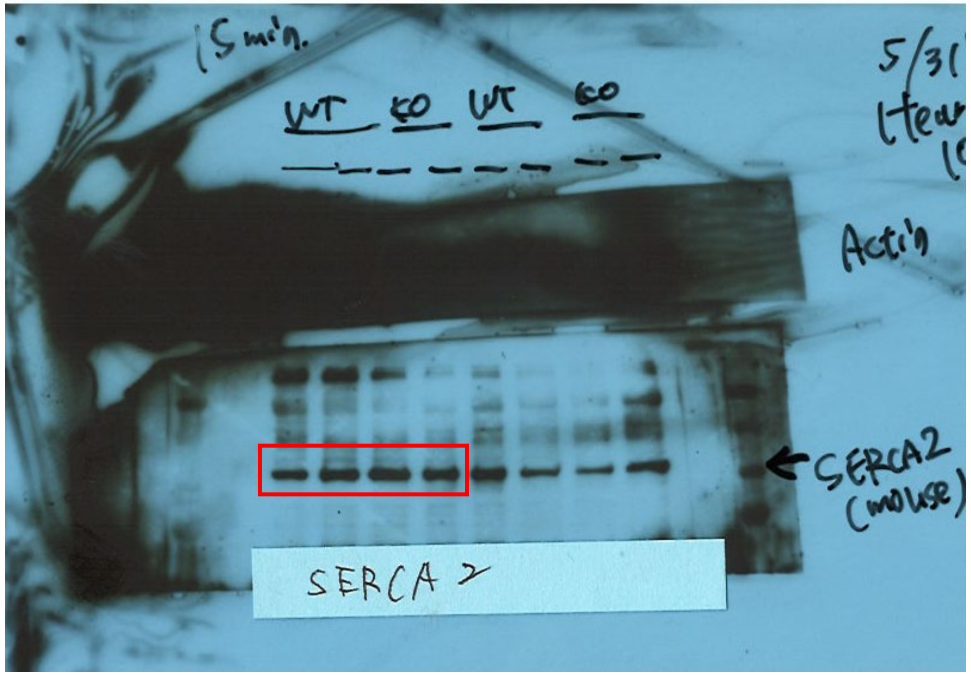

Figure 1. C – CSQs

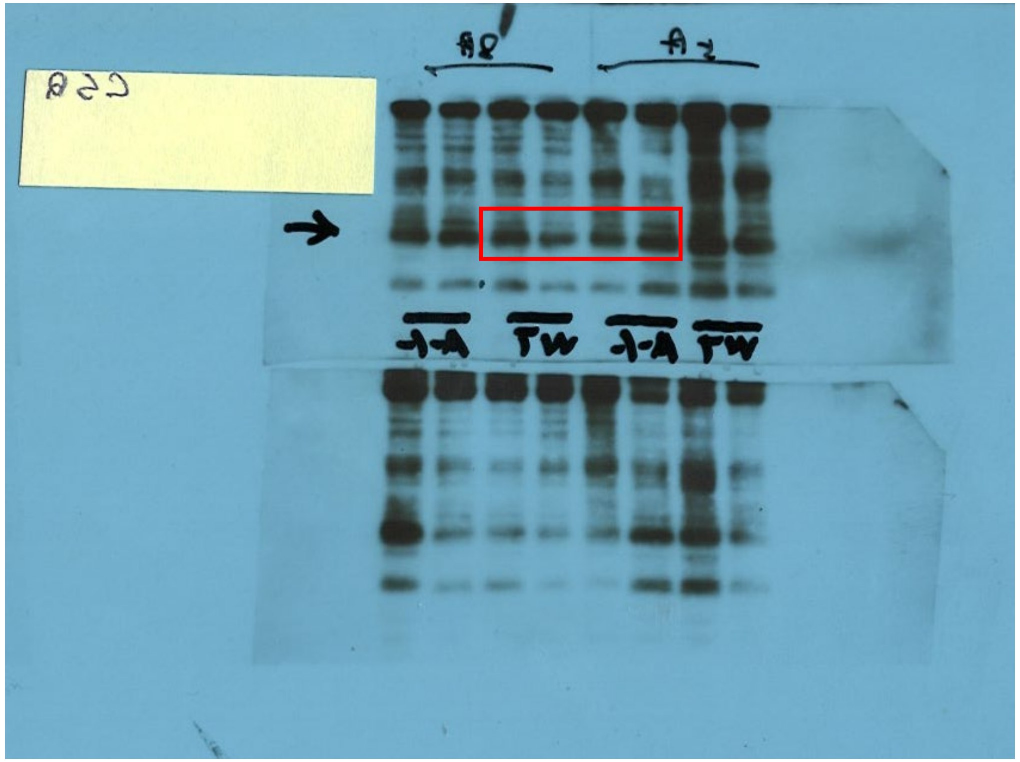

Figure 1. C – JP2

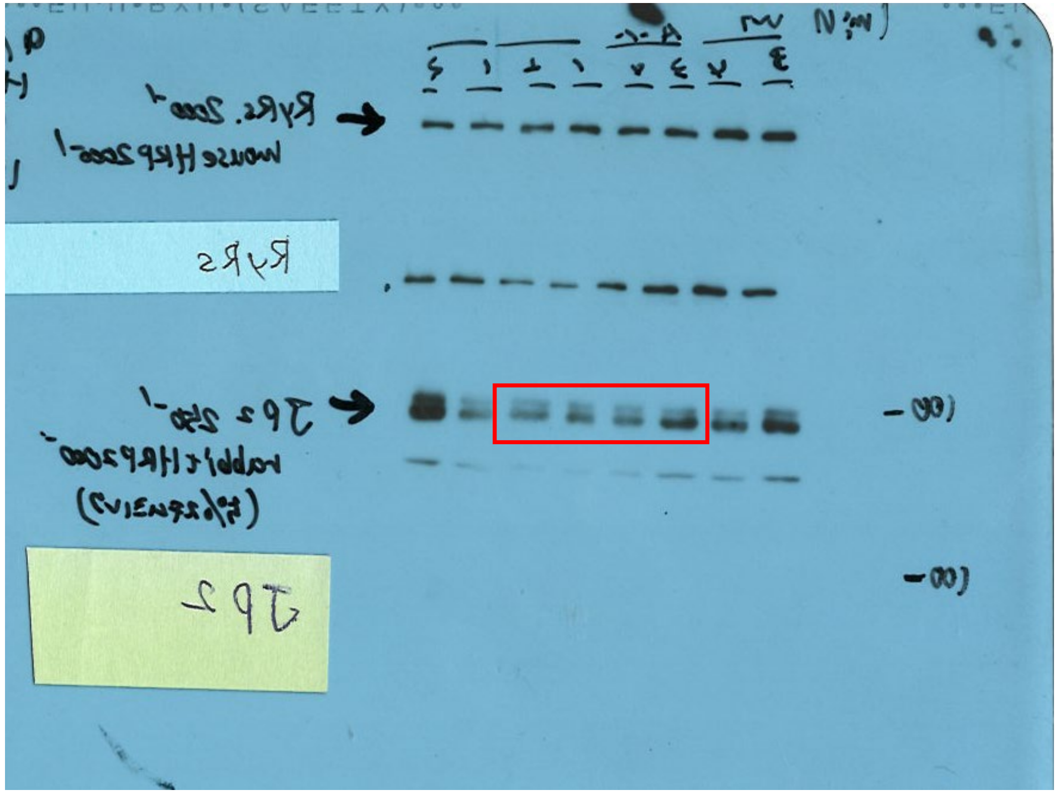

Figure 1. C – TRIC-A

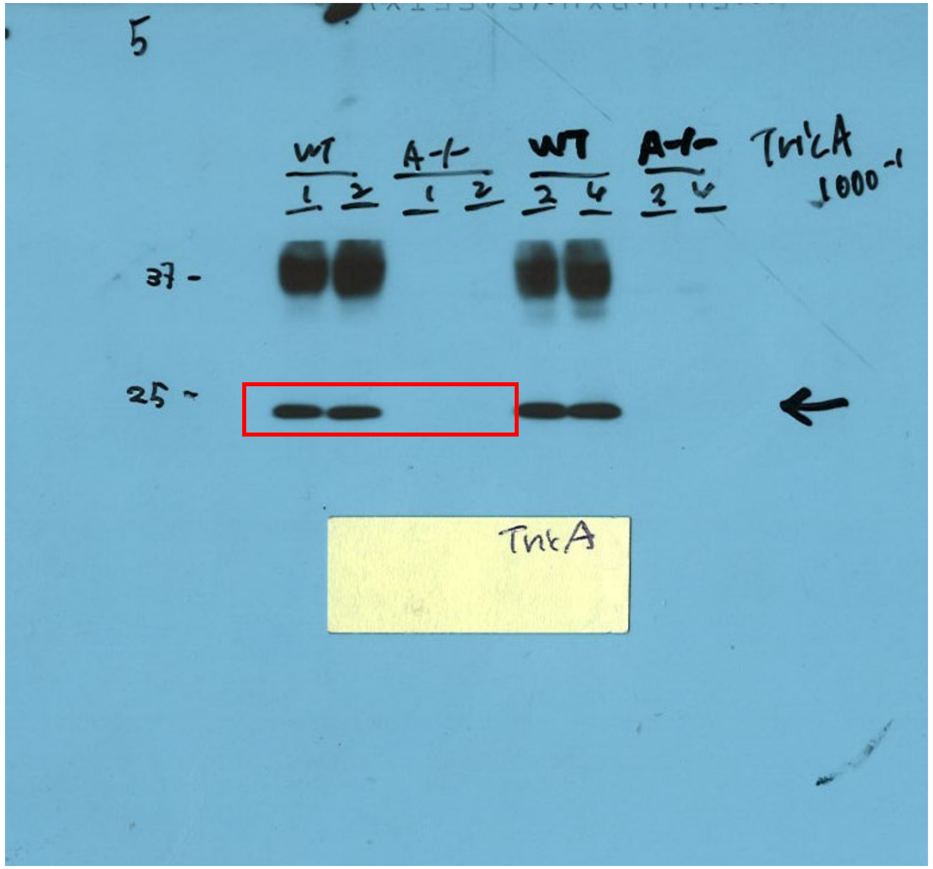

Figure 1. C – TRIC-B

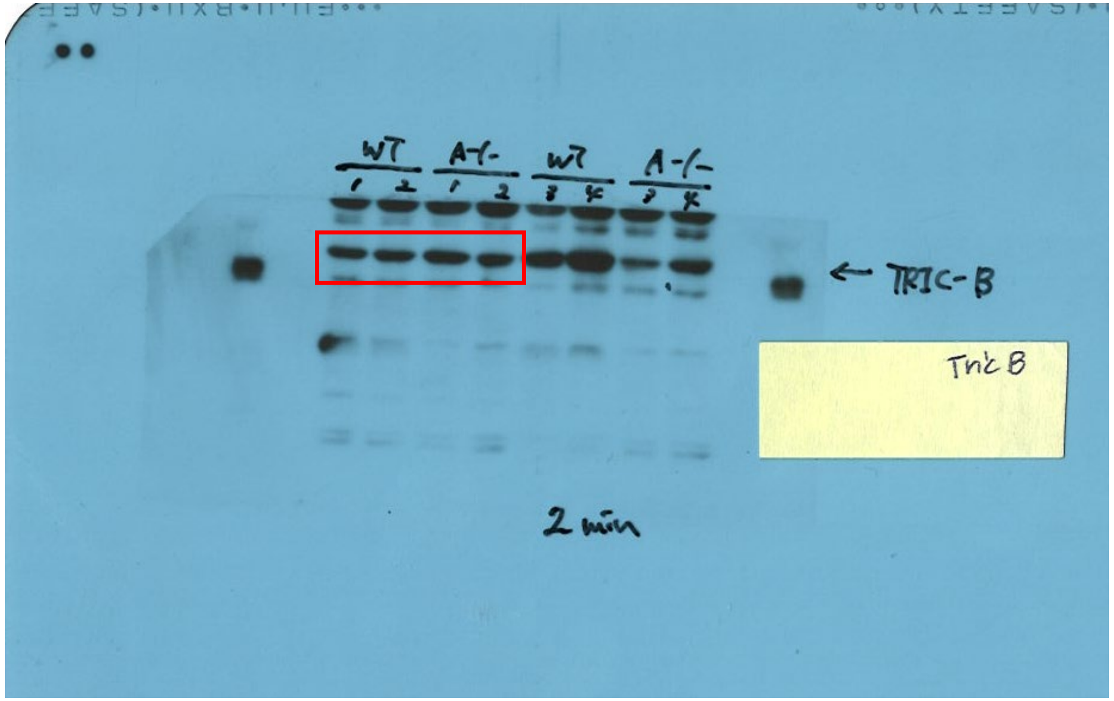

Figure 1. C – Actin

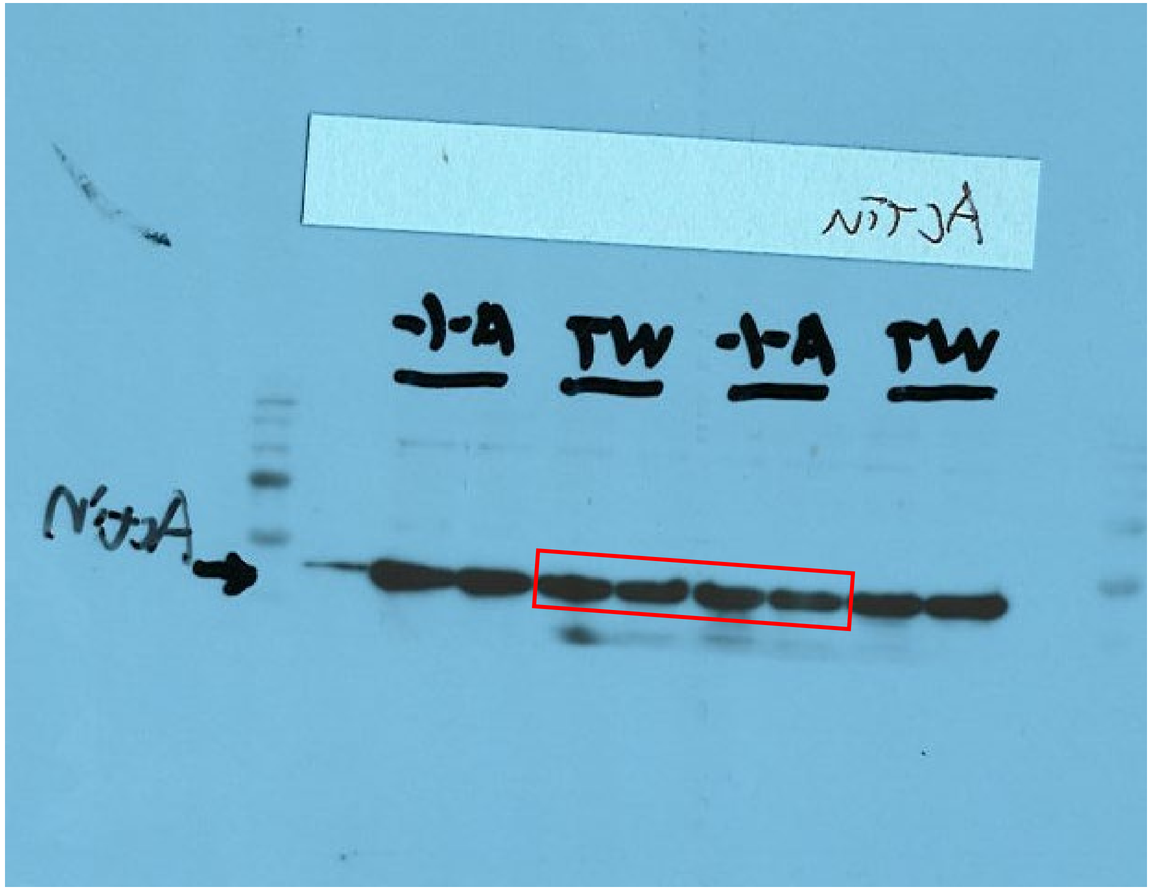

Figure 5. A – Myh6

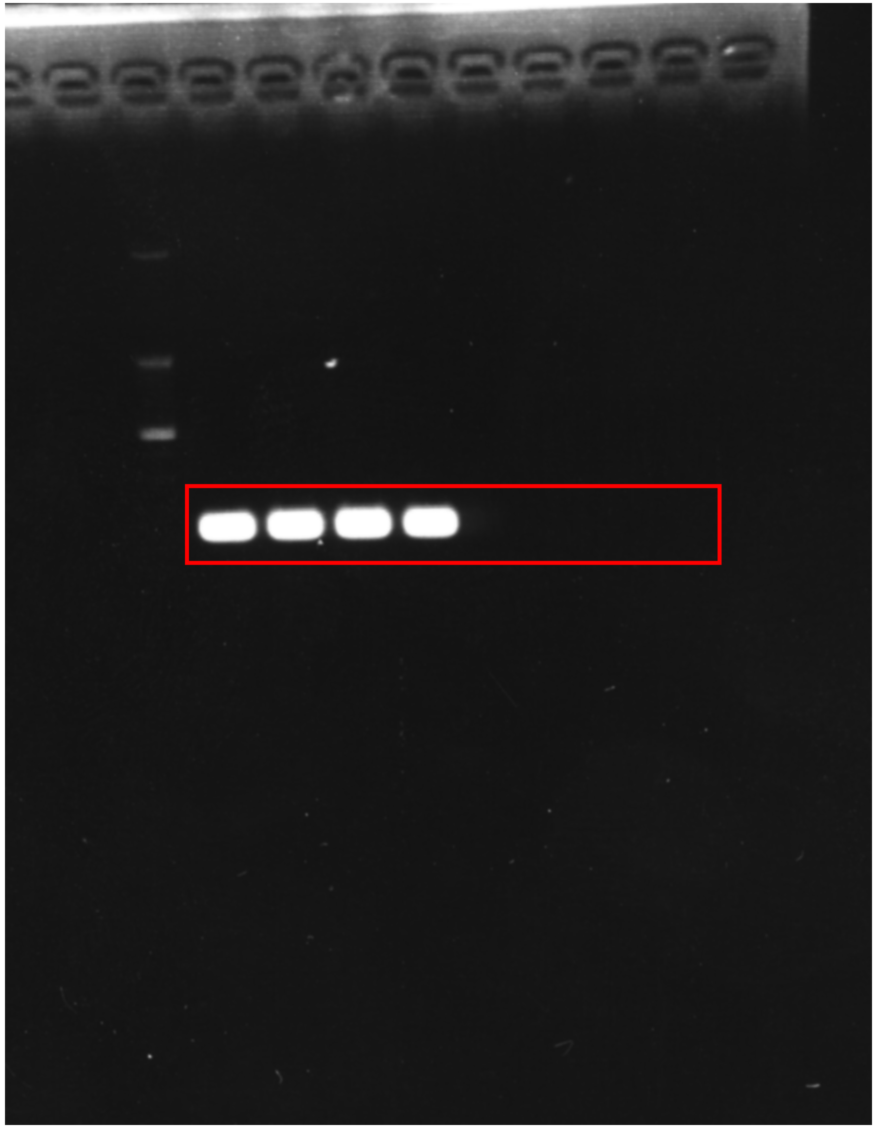

Figure 5. A – Ctgf

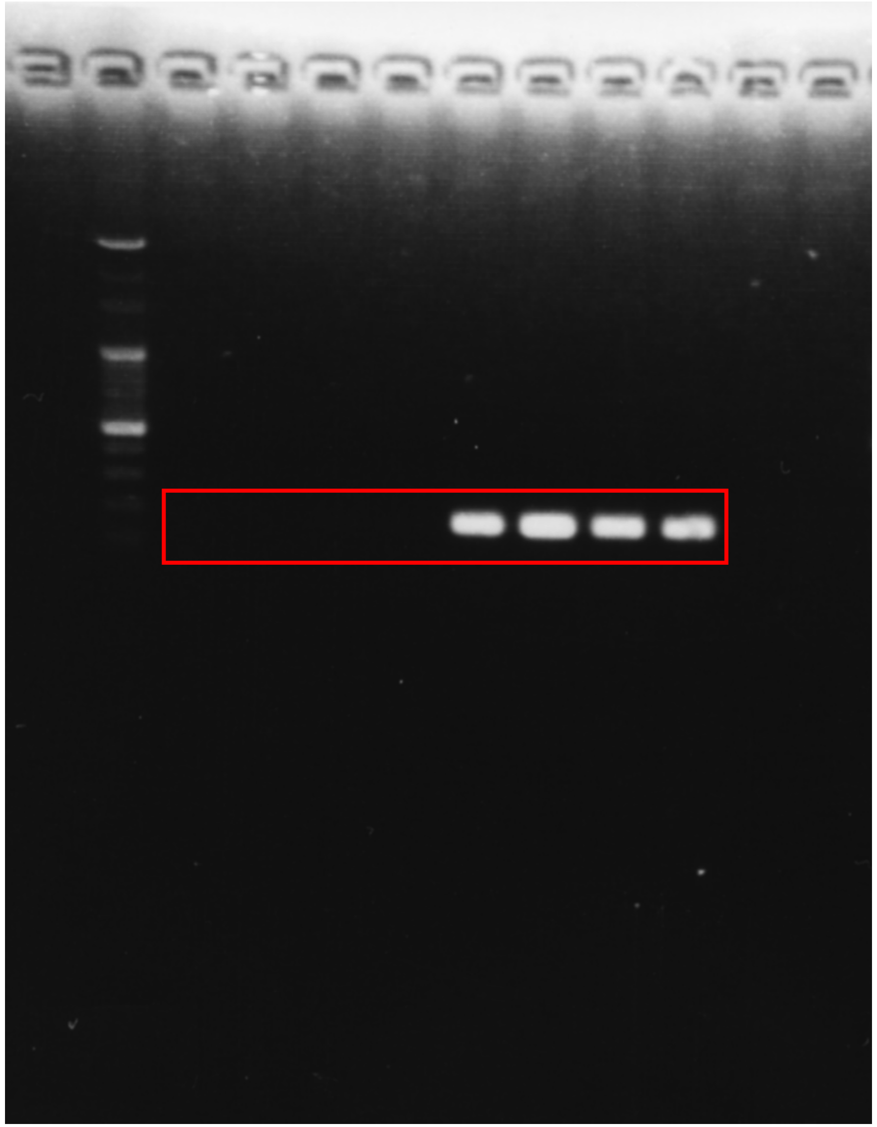

Figure 5. A – TRIC-A

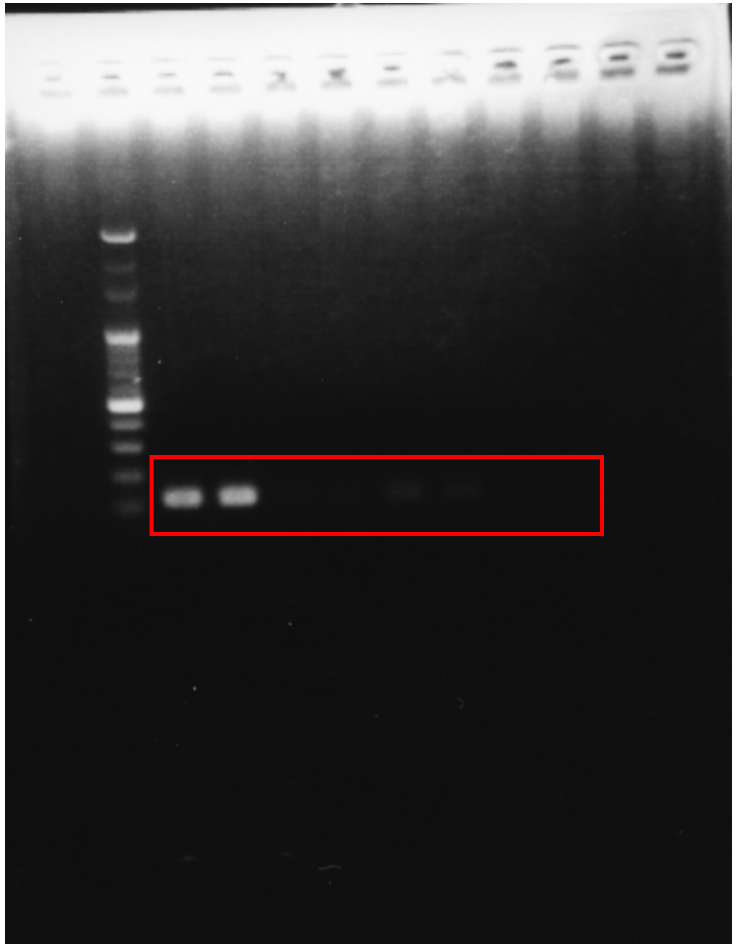

Figure 5. A – TRIC-B

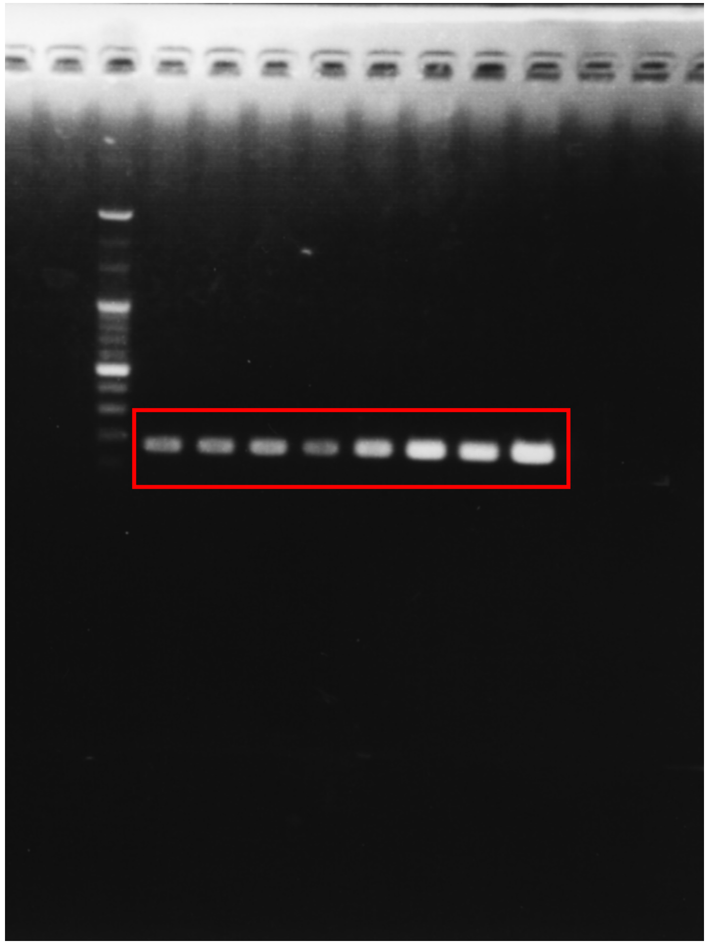

Figure 5. A – GAPDH

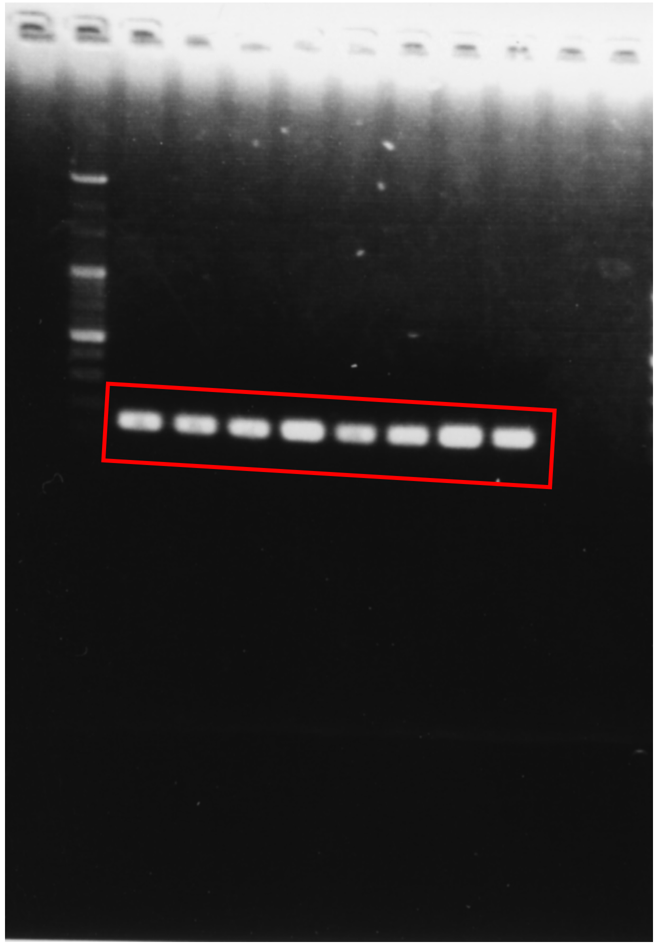

Supplement: Supplementary file 1 [file biomolecules-16-00181-s001.zip › biomolecules-4093635-supplementary.pdf]
